# Plasma apolipoprotein E levels in longitudinally followed patients with mild cognitive impairment and Alzheimer’s disease

**DOI:** 10.1186/s13195-022-01058-9

**Published:** 2022-08-24

**Authors:** Andreas Giannisis, Asma Al-Grety, Henrik Carlsson, Kalicharan Patra, Daniel Twohig, Sigrid Botne Sando, Camilla Lauridsen, Guro Berge, Gøril Rolfseng Grøntvedt, Geir Bråthen, Linda R. White, Kim Kultima, Henrietta M. Nielsen

**Affiliations:** 1 Department of Biochemistry and Biophysics, Stockholm University, Svante Arrhenius Väg 16B, 106 91, Stockholm, Sweden; 2 Department of Medical Sciences, Clinical Chemistry, Uppsala University, Uppsala, Sweden; 3 Department of Neurology, University Hospital of Trondheim, Trondheim, Norway; 4 Department of Neuromedicine and Movement Science (INB), Faculty of Medicine and Health Sciences, Norwegian University of Science and Technology (NTNU), Trondheim, Norway

**Keywords:** Apolipoprotein E, Plasma, Mass spectrometry, Alzheimer’s disease, *APOE* ε4

## Abstract

**Background:**

Low levels of plasma apolipoprotein E (apoE) and presence of the *APOE* ε4 allele are associated with an increased risk of Alzheimer’s disease (AD). Although the increased risk of AD in *APOE* ε4-carriers is well-established, the protein levels have received limited attention.

**Methods:**

We here report the total plasma apoE and apoE isoform levels at baseline from a longitudinally (24 months) followed cohort including controls (*n* = 39), patients with stable amnestic mild cognitive impairment during 24 months follow up (MCI-MCI, *n* = 30), patients with amnestic MCI (aMCI) that during follow-up were clinically diagnosed with AD with dementia (ADD) (MCI-ADD, *n* = 28), and patients with AD with dementia (ADD) at baseline (ADD, *n* = 28). We furthermore assessed associations between plasma apoE levels with cerebrospinal fluid (CSF) AD biomarkers and α-synuclein, as well as both CSF and plasma neurofilament light chain (NfL), YKL-40 and kallikrein 6.

**Results:**

Irrespective of clinical diagnosis, the highest versus the lowest apoE levels were found in *APOE *ε2/ε3 versus *APOE *ε4/ε4 subjects, with the most prominent differences exhibited in females. Total plasma apoE levels were 32% and 21% higher in the controls versus MCI-ADD and ADD patients, respectively. Interestingly, MCI-ADD patients exhibited a 30% reduction in plasma apoE compared to MCI-MCI patients. This decrease appeared to be associated with brain amyloid-β (Aβ_42_) pathology regardless of disease status as assessed using the Amyloid, Tau, and Neurodegeneration (A/T/N) classification. In addition to the association between low plasma apoE and low levels of CSF Aβ_42_, lower apoE levels were also related to higher levels of CSF total tau (t-tau) and tau phosphorylated at Threonine 181 residue (p-tau) and NfL as well as a worse performance on the mini-mental-state-examination. In MCI-ADD patients, low levels of plasma apoE were associated with higher levels of CSF α-synuclein and kallikrein 6. No significant correlations between plasma apoE and the astrocytic inflammatory marker YKL40 were observed.

**Conclusions:**

Our results demonstrate important associations between low plasma apoE levels, Aβ pathology, and progression from aMCI to a clinical ADD diagnosis.

**Supplementary Information:**

The online version contains supplementary material available at 10.1186/s13195-022-01058-9.

## Introduction

The human apolipoprotein E gene (*APOE*) is polymorphic and encodes three common apolipoprotein E (apoE) isoforms that differ in the presence or absence of cysteine (Cys) and arginine (Arg) residues at positions 112 and 158; apoE2: Cys112, Cys158, apoE3: Cys112, Arg158, and apoE4: Arg112, Arg158 [1–3]. Peripheral apoE isoforms are mainly derived from hepatocytes [4, 5] whereas apoE in the central nervous system (CNS) is predominantly secreted by glia cells [6–10], mainly astrocytes [11] and to a lesser extent by pericytes [12]. Under pathological conditions, neurons can also secrete apoE [13]. In both the peripheral and the CNS compartments, apoE plays an important role in mediating the transport of lipids between cells and tissues by interacting with receptors in the low-density lipoprotein receptor (LDLR) family. The small but important amino acid variations between the isoforms strongly influence their affinity to the LDLRs and their distribution in different lipoparticles [14].

Carriers of the *APOE* ε4 allele are at a 5 to 15-fold higher risk of developing Alzheimer’s disease (AD) [15] while the *APOE* ε3 allele is considered AD risk-neutral and ε2 protective [16, 17]. Although numerous studies have established a strong connection between AD and *APOE* ε4 [18], the apoE fluid levels have received little attention, especially plasma apoE since it cannot cross the blood–brain-barrier (BBB) [5]. The advance of studies on apoE levels, specifically in humans, is complicated by the very close similarity between the apoE isoforms hampering the use of immuno-based assays due to the lack of isoform-specific antibodies. Therefore, discrepancies in reported apoE levels are most likely due to method-related differences, supported by the very low correlation between results acquired by use of immuno-based enzyme-linked immunosorbent assay (ELISA) versus mass spectrometry (MS) [19]. Inconsistencies can be illustrated by findings showing that plasma apoE levels in AD patients were either unaltered [20, 21], decreased [22–24], or increased [25] compared to controls. Contradictory results have also been shown in studies assessing the levels of apoE in the cerebrospinal fluid (CSF) from AD patients compared to healthy individuals showing no differences [21, 26–28], decreased [29], or increased levels [30, 31]. We speculate that in addition to methodological bias, inconsistent results may further be due to modifiable variables attributed to specific cohorts, for example ethnicity and diet, and yet to be determined genetic factors.

Unrelated to disease status (AD or non-AD), we previously demonstrated that the *APOE* ε4 genotype is linked to reduced levels of plasma but not CSF apoE [21]. These findings are in line with those of other studies [20, 26]. Interestingly, low plasma apoE levels have been linked to lower hippocampal size [32], cognitive impairment [33], and an increased risk of AD and other types of dementia [24, 34]. The plasma apoE deficiency that we previously described in *APOE* ε4-carriers was specifically due to reduced levels of the apoE4 isoform, as assessed in *APOE* ε4 heterozygous individuals [21]. Why specifically the apoE4 levels are reduced in *APOE* ε4 heterozygotes is not clear, though higher turnover rates of apoE4 in plasma were previously reported [35]. Importantly, the distribution of the specific apoE isoforms in the total levels of apoE appear to be of significance. In a cohort of cognitively healthy *APOE ε*3/ε4 subjects, we found that a higher ratio of plasma apoE4 to apoE3 was linked to hippocampal glucose hypometabolism, an early feature in AD pathophysiology [36], and reduced grey matter volume in several brain areas implicated in AD [37]. Interestingly, the contribution of the different apoE isoforms to the total plasma apoE levels is not evenly distributed in plasma from *APOE* heterozygous individuals. By use of a selective reaction monitoring MS based assay [38], we have demonstrated that in individuals with an *APOE* ε2/ε3 and ε2/ε4 genotypes, the apoE2 isoform contributed to the total plasma apoE levels by 60% and 70% respectively. In *APOE* ε3/ε4 individuals, the apoE4 isoform accounted for only 30% of the total plasma apoE levels [21], whereas apoE3 and apoE4 isoforms in plasma from non-demented *APOE* ε3/ε4 subjects enrolled in the Arizona *APOE* cohort were very similar [37]. Results from other studies have confirmed that the apoE isoform ratio varies in individuals with different *APOE* heterozygous genotypes [20, 26].

In the current study, we utilized an MS-based apoE quantification method to confirm and to expand on our previously reported ELISA-acquired plasma apoE levels in *APOE* homozygous subjects [39]. We assessed the total plasma and apoE isoform levels in baseline samples from a longitudinally (24 months) followed cohort of patients with amnestic mild cognitive impairment (aMCI) or ADD, versus controls, and investigated potential associations with disease progression, cognition, AD pathology as assessed using the Amyloid, Tau and Neurodegeneration (A/T/N) classification, and CSF AD biomarkers, α-synuclein, neurofilament light chain (NfL), YKL-40 and kallikrein 6 (KLK6) levels in CSF or plasma.

## Materials and methods

### Participants

Study participants (*n* = 125) were ethnic Norwegians enrolled at the Department of Neurology, University Hospital, Trondheim, Norway, between 2009 and 2015, and followed over a period of 24 months. Inclusion criteria and diagnostic and descriptive data were previously reported [40]. At baseline, the subjects were healthy individuals (*n* = 39) and patients with an aMCI (*n* = 58) or ADD (*n* = 28) diagnosis. Plasma was obtained from all the subjects after centrifugation of blood samples (1500 × g, 10 min, room temperature) collected in ethylenediaminetetraacetic acid (EDTA) containing tubes. Following repeated clinical assessments, aMCI patients either remained stable (MCI-MCI, *n* = 30) or were diagnosed with ADD (MCI-ADD, *n* = 28) at 24 months follow-up. The aMCI or ADD diagnoses at baseline were given by clinicians blinded to CSF biomarkers and according to the International Working Group on Mild Cognitive Impairment criteria or NINCDS-ADRDA criteria respectively [41, 42], without considering CSF biomarkers cut-offs. *APOE* genotype, CSF sampling, and assessment of CSF AD biomarkers levels including amyloid-β42 (Aβ_42_), amyloid-β40 (Aβ_40_), total tau (t-tau), and phosphorylated tau at Threonine (Thr) 181 (p-tau) allowing for A/T/N classification, along with other markers including CSF α-synuclein and NfL, CSF, and plasma levels of KLK6, were previously reported [15, 40, 43–47].

### Sample preparation for liquid chromatography − mass spectrometry analysis

In a 96-well plate (MicroAmp™ Optical 96-Well Reaction Plate, ThermoFisher Scientific, Waltham, MA, USA), 15 μL of plasma, diluted 1:100 with ammonium bicarbonate 50 mM (approximately 10 μg of total protein), was denatured with 0.05% RapiGest SF Surfactant (Waters Corporation, Milford, MA, USA) and then reduced at 60 °C for 45 min with 10 mM dithiothreitol (DTT, Sigma Aldrich, St. Louis, MO, USA). Further, samples were incubated at room temperature in the dark for 40 min with 30 mM iodoacetamide (IAA, Sigma Aldrich) for alkylation of the Cys residues. Excess IAA was quenched with the addition of 20 mM DTT and incubation for 15 min at room temperature. Subsequently, a mixture of heavy labeled peptides corresponding to endogenous apoE peptides LGADMEDVCGR common for both apoE2 and apoE3 isoforms, LGADMEDVR present only in apoE4 isoform, LAVYQAGAR common for both apoE3 and apoE4 isoforms, CLAVYQAGAR present only in apoE2 isoform, and LGPLVEQGR which is common to all three apoE isoforms (SpikeTides TQL, JPT Peptide Technologies GmbH, Berlin, Germany Supplementary Table 1) was added to each sample and the samples were further digested using two consecutive digestion steps with trypsin (0.1 mg/mL, ThermoFisher Scientific) as well as a mixture of trypsin/lysine C (0.1 mg/mL, ThermoFisher Scientific), each in a ratio 1/20 (μg of enzyme/μg of protein). Samples were first incubated with trypsin for 4 h at 37 °C, followed by an incubation with trypsin and trypsin/lysine C for 18–19 h at 37 °C. The next day, samples were treated with 1% trifluoroacetic acid (TFA) and centrifuged for 15 min at 17,000 × g to precipitate the RapiGest SF Surfactant. The supernatant was collected, and the digested peptides were cleaned and extracted by solid phase extraction using an Oasis hydrophilic-lipophilic balance (HLB) 96-well μElution Plate with 2 mg Sorbent per well, 30 μm particle size (Waters Corporation). The clean-up of endogenous as well as exogenous heavy labeled peptides was performed according to the supplier’s recommendations with minor modifications; the plate wash was performed with MiliQ water, and the peptides were eluted with 25 μL 100% methanol. Eluted peptides were dried under vacuum and stored at –80 °C until analysis.

### Liquid chromatography − mass spectrometry analysis

Liquid chromatography (LC) (Dionex UltiMate™ 3000 RSLC Nano, ThermoFisher Scientific) was used for separation of the plasma apoE isoforms, whereas detection and quantification of the resulting tryptic peptides was performed using a Q Exactive Orbitrap mass spectrometer (ThermoFisher Scientific) operated at single stage full scan, essentially as described before [37, 38]. Briefly, the day of the analysis, dried peptides were re-suspended in 30 μL 0.1% formic acid in MiliQ water. The solubilized samples were transferred to 0.3 mL polypropylene Snap Ring Micro-Vials (32 × 11.6 mm, Genetec, Montréal, Canada) and sealed with polyethylene Snap Ring caps with a center hole (11 mm, Genetec). Five microliters from each sample were injected on a reversed phase, C18 trap column (5 × 0.3 mm, 5 μm, ThermoFisher Scientific), and further eluted from a PepMap C18 analytical column (150 × 0.15 mm, 2 μm, ThermoFisher Scientific) coupled to an Easy Spray source (ThermoFisher Scientific). The temperature of the analytical column was 40 °C, and the flow rate was 1 μL/min. Peptides were eluted over an 11-min gradient with the concentration of the mobile phase B (0.1% formic acid in acetonitrile) increasing over the mobile phase A (0.1% formic acid in MiliQ water) to a maximum 40%. The scan range was m/z 470 – 621, the resolution was 70,000, the automatic gain control (AGC) target was set at 1 × 10^6^, and the maximum injection time was 100 ms.

### Plasma apoE quantification

For apoE isoform quantification, the spiked amount of each heavy peptide (Supplementary Table 1) was multiplied with the area response ratio between the endogenous peptide to the corresponding heavy peptide. The obtained value was multiplied by the dilution factor (100x) and divided by fifteen to account for the diluted plasma sample volume, corresponding to the concentration of the plasma apoE in fmoles/μL which was converted to μg/mL using the apoE molecular weight (34 kDa) (Supplementary Fig. 1a). Levels of absolute plasma apoE were determined by the recorded area of the common apoE peptide LGPLVEQGR (Supplementary Fig. 1a), as well as by specific peptides for each isoform (Supplementary Table 2). More specifically, in individuals with the *APOE* ε3/ε3, *APOE* ε3/ε4, and *APOE* ε4/ε4 genotype, the area response of the peptide LAVYQAGAR was used for the quantification of apoE3, total apoE3/4, and apoE4 levels according to formula in the Supplementary Fig. 1a, because the obtained concentrations showed excellent linearity with the peptide LGPLVEQGR (Supplementary Fig. 1b). Levels of apoE3 in subjects with *APOE* ε3/ε4 genotype were determined by the peptide LGADMEDVCGR, while the amount of the apoE4 isoform was calculated by subtracting the amount of the apoE3 peptide LGADMEDVCGR from the common apoE3/4 peptide LAVYQAGAR amount. The apoE4 isoform-specific peptide LGADMEDVR was used only for assessing the apoE phenotype and not for quantification due to sample variability (Supplementary Fig. 1c-d). In *APOE* ε2 carriers, the levels of apoE2 were determined by use of the formula illustrated in Supplementary Fig. 1a using the area response for the peptide CLAVYQAGAR, whereas the observed area for the peptide LAVYQAGAR was used for the determination of apoE3 and apoE4 isoform levels according to the same formula (Supplementary Fig. 1a). Lastly, total plasma apoE levels as determined by the common apoE peptide were compared to and validated by the sum of the individual apoE isoforms levels (Supplementary Fig. 1e). The linearity range for the quantification of apoE isoforms was assessed in calibration curves for the corresponding peptides by spiking known amounts of the heavy peptide (3.1–821 fmoles) into a plasma pool that contained samples from subjects with the *APOE* ε2/ε4, ε3/ε3, ε2/ε3, and ε4/ε4 genotypes. In the sample pool, each genotype was represented by plasma from one male and one female mixed in a 1:1 ratio (total 8 samples). Calibration curves were generated using 1/X^2^ weighting (Supplementary Fig. 2). The detected amounts of apoE peptides used for quantification ranged between 3.7 and 612 fmoles. Averaged intra-assay coefficients of variance (CV%) for the peptides LGPLVEQGR, LAVYQAR, and CLAVYQAGAR were < 3%, whereas for the peptide LGADEMDVCGR, the CV % was < 7%. For all peptides, the inter-assay variation was < 15%.

### Plasma NfL analysis

Levels of plasma NfL were determined in plasma samples from *n* = 123 individuals using standardized service protocols and the Simoa™ NF-light® Kit (Quanterix) at PBL Assay Science (Piscataway, NJ, USA). Intra- and inter-assay variations were 4% and 9% respectively.

### Plasma and CSF YKL-40 analyses

Levels of YKL-40 were assessed in plasma and CSF samples from *n* = 125 and *n* = 120 individuals diluted 1:200 and 1:4 respectively in phosphate buffered saline (PBS) containing 1% bovine serum albumin (BSA). Plasma levels of YKL-40 were determined using the Human Chitinase 3-like 1 DuoSet ELISA kit (R&D systems, MN, USA), while for the determination of CSF YKL-40, the MicroVue YKL-40 EIA ELISA kit (Quidel, San Diego, CA, USA) was utilized, following the suppliers’ guidelines. Plasma and CSF intra-assay variations were 3% and 4% respectively, whereas the inter-assay variation for plasma was 8% and CSF was 11%. Recovery percentage for plasma was between 69% and 96%, whereas for CSF it was between 65% and 79%.

### Data analysis

For the apoE analysis, the chromatographic spectra were analyzed by TraceFinder version 5.1 (ThermoFisher Scientific). Calibration curves were generated using the GraphPad Prism, version 9 (GraphPad Inc., La Jolla, CA, USA). Statistical analyses were performed using JMP Pro statistical software, version 15.0.0 (SAS Institute, NC, USA) and the IBM SPSS Statistics 28. Normal distribution was assessed by using the Kolmogorov–Smirnov test for normality, while non-normally distributed variables were log-transformed, and the distribution was re-assessed. Group comparisons (≥ 3 groups) were performed using analysis of variance (ANOVA, post hoc Tukey HSD) or the Kruskal–Wallis test (post hoc Dunn’s test) when log transformation did not result in normally distributed data. Depending on the data distribution, comparisons of results between two groups were performed using the Student’s *t*-test or the Mann–Whitney *U* test. Group comparisons for variables significantly associated with age (CSF YKL-40, plasma and CSF NfL) were performed using analysis of covariance (ANCOVA) or Quade nonparametric ANCOVA depending on the data distribution. Assessment of the effect of the *APOE* genotype on the group comparisons of plasma apoE between diagnostic groups was performed using linear regression model with 2 dummy variates (0 and 1), for each *APOE* genotype. Significance of the model was assessed using Wald Chi-Square. Bonferroni correction was used to account for multiple comparisons (*n*), where applicable. Associations between variables were assessed utilizing the Pearson’s (*r*) test, or the Spearman’s (*ρ*) test, or partial correlations (*r*(degrees of freedom)) controlling for *APOE* genotype, depending on the data distribution. Results are presented as average ± standard deviation or median (minimum – maximum). A *p*-value of ≤ 0.05 was considered statistically significant.

## Results

### Study cohort demographics and clinical characteristics

The demographic and clinical characteristics of subjects included in the parent cohort have been published elsewhere [39, 40, 44, 45]. Characteristics specific to the now included subjects are summarized in Table 1. Briefly, controls were 5 years older compared to MCI-ADD (*p* < 0.001) and ADD patients (*p* < 0.001), and as expected, the controls exhibited the highest MMSE test scores, the highest CSF Aβ_42_, Aβ_40_ levels, and the lowest t-tau and p-tau levels (Table 1). As expected, controls exhibited a higher ratio of CSF Aβ_42_ over Aβ_40_ (Aβ_42_/Aβ_40_) compared to MCI-ADD (*p* = 0.001) and ADD (*p* < 0.001) patients. Among the controls, the frequency of the *APOE* ε4 allele (41%) was significantly lower compared to the MCI-MCI (63%), MCI-ADD (75%) and ADD groups (86%) (chi-square, *p* = 0.001) (Table 1). Based on a recent assessment of the parent cohort [40], CSF AD biomarker cut-off levels (630 pg/mL for Αβ_42_ [47], 66 pg/mL for p-tau and 394 pg/mL for t-tau) were established to allow for classification of the included subjects according to the A/T/N classification system (Table 2). Levels of CSF α-synuclein and KLK6 levels in both plasma and CSF have been described elsewhere [15, 43–45] and used only for correlation analyses in the current study.

**Table 1:** Demographics and clinical characteristics

	*N* (F/M)	*APOE* ε4 allele status (− / − , + / − , + / +)	Age (years)	MMSE score	CSF Aβ_42_ (pg/mL)	CSF Aβ_40_ (pg/mL)	CSF Aβ_42_/Aβ_40_	CSF t-tau (pg/mL)	CSF p-tau (pg/mL)
Whole cohort	125 (71/54)	45, 50, 30	65.0 (53.0–84.0)	28.0 (16.0–30.0)	602.6 (173.0–1674.1)	15,387.0 (3553.0–33,373.5)	0.045 (0.010–0.120)	395.5 (98.5–2325.3)	67.4 (15.9–168.8)
Controls	39 (26/13)	23, 16, 0	69.0 (57.0–84.0)	29.0 (28.0–30.0)	1010.7 (628.8–1674.1)	16,635.0 (11,152.0–33,373.5)	0.062 (0.020–0.100)	264.1 (137.5–558.1)	53.5 (32.8–102.0)
MCI-MCI	30 (14/16)	11, 8, 11	64.0 (53.0–79.0)	28.0^**^ (25.0–30.0)	591.5^***^ (173.0–1268.8)	13,477.0^*^ (3553.0–31,343.0)	0.048 (0.010–0.120)	319.8 (98.5–1057.0)	55.7 (15.9–131.0)
MCI-ADD	28 (17/11)	7, 10, 11	63.5^***^ (56.0–71.0)	27.0^***^ (23.0–29.0)	526.7^***^ (282.7–1059.8)	13,667.5 (8021.0–23,258.8)	0.037*** (0.010–0.090)	557.9^***^ (163.0–2325.3)	86.3^***^ (37.3–168.8)
ADD	28 (14/14)	4, 16, 8	64.0^***^ (54.0–78.0)	23.0^***^ (16.0–27.0)	481.6^***^ (211.6–1092.2)	15,387.0 (6708.0–29,090.0)	0.034*** (0.010–0.080)	611.8^***^ (176.5–1540.0)	90.2^***^ (27.9–156.9)
*p*-value	ns^a^	< 0.001^a^	< 0.001^b^	< 0.001^b^	< 0.001^c^	0.035^c^	< 0.001^b^	< 0.001^c^	< 0.001^c^

Results are represented as median (minimum–maximum)

*MCI-MCI* patients with amnestic mild cognitive impairment that did not fulfil the ADD diagnostic criteria upon 24 months follow-up, *MCI-ADD* patients with amnestic mild cognitive impairment that fulfilled the AD diagnostic criteria after 24 months, *ADD* patients with Alzheimer’s disease with dementia at baseline, *F* Females, *M* Males, *APOE* Apolipoprotein E gene, *MMSE* Mini mental state examination, *Aβ*_*42*_ Amyloid-β42 peptide, *Αβ*_*40*_ Amyloid-β40 peptide, *t-tau* Total tau, *p-tau* Tau phosphorylated at Threonine 181 residue, *ns* non-significant, *a* chi-square test, *b, c* multiple comparison of the diagnostic groups using Kruskal–Wallis (b) or ANOVA (c)

^*^p ≤ 0.05, **p ≤ 0.01, and ***p ≤ 0.001 indicate comparison of the patient groups with the control group using Tukey HSD or Dunn’s test followed by Bonferroni correction for multiple comparisons (*n* = 6)

**Table 2:** A/T/N classification

	A-/T-/N- (*n*)	A-/T-/N + (*n*)	A-/T + /N- (*n*)	A-/T + /N + (*n*)	A + /T-/N- (*n*)	A + /T-/N + (*n*)	A + /T + /N- (*n*)	A + /T + /N + (*n*)
Whole cohort (*n* = 123)	37	2	6	13	15	3	2	45
Controls (*n* = 38)	26	-	5	5	-	-	-	2
MCI-MCI (*n* = 29)	8	1	1	3	7	1	-	8
MCI-ADD (*n* = 28)	2	-	-	3	3	1	2	17
ADD (*n* = 28)	1	1	-	2	5	1	-	18

Amyloid/Tau/Neurodegeneration (A/T/N) classification based on previously reported cut-off levels of CSF AD biomarkers Αβ_42_, p-tau and t-tau [40]; (A +) Αβ_42_ < 630 pg/mL [47], (T +) p-tau > 66 pg/mL and (N +) t-tau > 394 pg/mL [40]. *MCI-MCI* patients with amnestic mild cognitive impairment that did not fulfil the ADD diagnostic criteria after 2 years, *MCI-ADD* patients with amnestic mild cognitive impairment that progressed to fulfil the ADD diagnostic criteria after 2 years follow-up, *ADD* patients with Alzheimer’s disease with dementia at baseline

### Plasma and CSF NfL and YKL-40 levels

Plasma NfL and YKL-40 and CSF YKL-40 levels are presented in Table 3, whereas CSF NfL levels were previously reported [46]. Globally, CSF NfL levels were nearly 60 times higher than in the plasma and the levels in both compartments positively correlated (Spearman’s (*ρ*) = 0.323, *p* = 0.002, *n* = 92) and also significantly associated with age (plasma NfL: Spearman (*ρ*) = 0.220, *p* = 0.015, *n* = 123, CSF NfL: Spearman’s (*ρ*) = 0.229, *p* = 0.026, *n* = 94). Similarly, plasma and CSF levels of YKL-40 were positively correlated (Spearman’s (*ρ*) = 0.404, *p* < 0.001, *n* = 120), but only CSF YKL-40 was significantly associated with age (plasma YKL-40; Spearman’s (*ρ*) = 0.150, *p* = 0.094, *n* = 125, and CSF YKL-40: Pearson’s (*r*) = 0.378, *p* < 0.001, *n* = 120). Neither plasma nor CSF NfL and YKL-40 levels were influenced by *APOE* ε4 status (plasma NfL: Kruskal–Wallis, *p* = 0.575, Plasma YKL-40: ANOVA, *p* = 0.794, CSF NfL: ANOVA, *p* = 0.175, CSF YKL-40: ANOVA, *p* = 0.855).

**Table 3:** Plasma and CSF NfL and YKL-40 levels

Plasma NfL (pg/mL)		Plasma YKL-40 (ng/mL)		CSF YKL-40 (ng/mL)	
Whole cohort (*n* = 123)	18.53 (6.98–216.57)	Whole cohort (*n *= 125)	45.1 (8.4–355.1)	Whole cohort (*n *= 120)	272.3 (102.4–538.7)
Controls (*n* = 39)	17.03 (6.98–216.57)	Controls (*n* = 39)	44.9 (13.0–316.3)	Controls (*n* = 37)	307.1 (102.4–506.1)
MCI-MCI (*n* = 29)	17.60 (7.34–45.82)	MCI-MCI (*n* = 30)	49.7 (8.4–168.7)	MCI-MCI (*n* = 28)	233.6 (126.9–507.2)
MCI-ADD (*n* = 27)	19.57 (7.59–42.38)	MCI-ADD (*n* = 28)	44.9 (16.3–248.4)	MCI-ADD (*n* = 28)	307.8 (156.2–538.7)
ADD (*n* = 28)	20.81 (12.61–94.97)^*, #^	ADD (*n* = 28)	45.4 (15.8–355.1)	ADD (*n* = 27)	260.2 (161.4–519.4)
*p*-value	0.008^a^	*p*-value	ns^b^	*p*-value	ns^c^

Results are presented as median (minimum–maximum). *MCI-MCI* patients with amnestic mild cognitive impairment that did not progress to an ADD diagnosis during the study period, *MCI-ADD* patients with amnestic mild cognitive impairment that fulfilled the ADD diagnostic criteria after two years, *ADD* patients with Alzheimer’s disease with dementia at baseline, NfL: Neurofilament light chain, *ns* Non-significant, *a* Quade nonparametric ANCOVA, *b* Kruskal–Wallis, *c* ANCOVA with age as covariant

^*^,^#^: *p* ≤ 0.05 corresponds to pairwise comparison between ADD patients and controls (*) or MCI-MCI (^#^) corrected for multiple comparisons (*n* = 6)

Accounting for the significant age difference between sexes (females: 65.0 ± 4.8, males: 67.6 ± 6.9, Student’s *t*-test, *p* = 0.014) as well as between the controls and the patients, CSF and plasma NfL as well as CSF YKL-40 group comparisons were performed with age as a covariant (ANCOVA, or Quade nonparametric ANCOVA). Between the sexes, levels of YKL-40 in plasma (Mann–Whitney *U* test, *p* = 0.656) and CSF (ANCOVA, *p* = 0.631), as well as NfL in plasma (Quade nonparametric ANCOVA, *p* = 0.290), were not different; however, males exhibited 6% higher CSF NfL levels compared to females (ANCOVA, *p* < 0.001).

Between the diagnostic groups, baseline ADD patients exhibited 1.2-fold higher plasma NfL levels compared to controls or MCI-MCI patients (Table 3). Comparison between controls, stable MCI, and ADD (baseline and MCI-ADD) revealed the same results (Quade nonparametric ANCOVA, *p* = 0.006, post hoc with Bonferroni adjustment for *n* = 3, ADD vs controls: *p* = 0.042, ADD vs stable MCI: *p* = 0.042). Levels of YKL-40 in plasma and CSF did not differ between the diagnostic groups (plasma: Kruskal–Wallis, *p* = 0.927, CSF: ANCOVA, *p* = 0.127) (Table 3).

### ApoE phenotype confirmation

In all the samples, the apoE phenotype was assessed based on the presence or absence of endogenous variants specific to each *APOE* genotype (Supplementary Table 2), as previously described [38]. The acquired apoE phenotypes (Table 4) were compared to the previously assessed *APOE* genotype and were in 100% accordance.

**Table 4:** ApoE phenotype determined by mass spectrometry

Diagnosis	*APOE* ε2/ε3 (*n*)	*APOE* ε2/ε4 (*n*)	*APOE* ε3/ε3 (*n*)	*APOE* ε3/ε4 (*n*)	*APOE* ε4/ε4 (*n*)
Whole cohort (*n* = 125)	5	3	40	47	30
Controls (*n* = 39)	2	2	21	14	0
MCI-MCI (*n* = 30)	3	1	8	7	11
MCI-ADD (*n* = 28)	0	0	7	10	11
ADD (*n* = 28)	0	0	4	16	8

*MCI-MCI* patients with amnestic mild cognitive impairment that did not progress to an ADD diagnosis, *MCI-ADD* patients with mild cognitive impairment that progressed to ADD diagnosis after two years, *ADD* Alzheimer’s disease with dementia patients diagnosed at baseline, *APOE* Apolipoprotein E gene

### *APOE* ε4 associated with lower plasma apoE levels

**Fig. 1:**
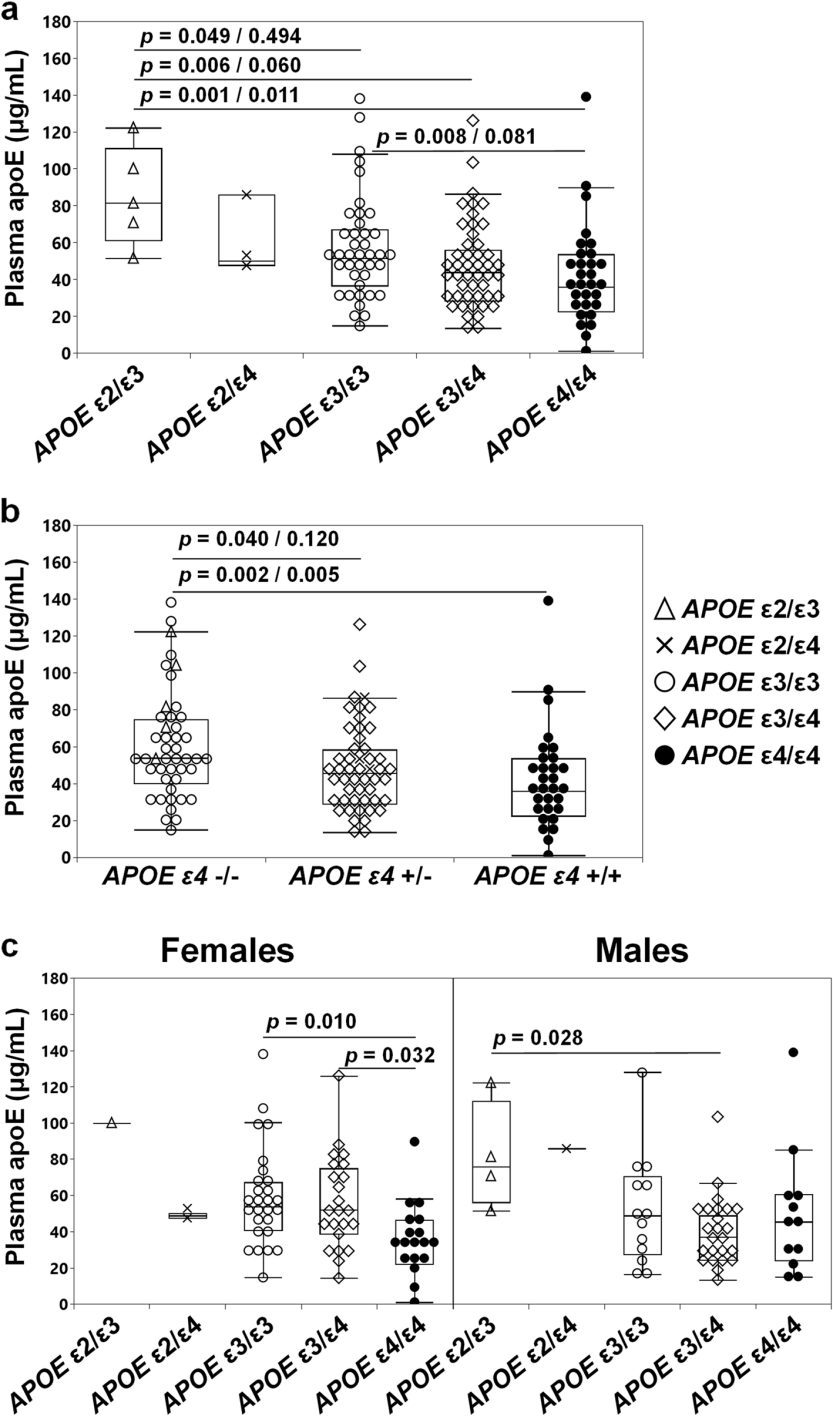
Levels of plasma apoE in subjects with different *APOE* genotypes. Plasma apoE levels as assessed in subjects grouped based on their *APOE* genotype (**a**), *APOE* ε4 status (**b**), and in males and females with different *APOE* genotype (**c**). Data are shown as median (minimum–maximum). Group comparisons were done using the Kruskal–Wallis test followed by Dunn’s test (**a**, **b**) before/after Bonferroni correction for multiple comparisons or ANOVA with Tukey HSD as post hoc test (**c**)

Previous studies have repeatedly shown that presence of the ε4 allele is linked to lower plasma levels of apoE [20, 21]. Comparing levels of plasma total apoE across the five *APOE* genotypes present in the current study we also recorded *APOE* genotype-specific effects on plasma apoE levels (Kruskal–Wallis, *p* = 0.004). As shown in Fig. 1a, levels of plasma total apoE were the highest in *APOE* ε2/ε3 subjects and the lowest in individuals with the ε4/ε4 genotype. In more detail, plasma apoE levels in *APOE* ε4/ε4 carriers were 30% and 56% lower compared to individuals with the ε3/ε3 and ε2/ε3 genotype (Fig. 1a). Plasma apoE levels were directly associated with *APOE* ε4 allele dose (zero, one or two copies) (Kruskal–Wallis, *p* = 0.006) and plasma apoE in heterozygous and homozygous individuals were 16% versus 33% lower compared to ε4 non-carriers (Fig. 1b). The latter difference remained significant after accounting for multiple comparisons (*n* = 3).

### Plasma total apoE levels and effects of sex

With female sex as a strong risk factor for AD [48, 49], we examined whether sex was associated with variations in plasma apoE levels. Due to low sample numbers, we excluded females with the *APOE* ε2/ε3 (*n* = 1) and ε2/ε4 (*n* = 2) genotype, as well as males with the ε2/ε4 (*n* = 1) genotype from the group comparisons. Levels of total plasma apoE were different across females with *APOE* ε3/ε3 (*n* = 27), ε3/ε4 (*n* = 23), and ε4/ε4 (*n* = 18) (ANOVA, *p* = 0.009), with ε4/ε4 females exhibiting the lowest levels. Among males with the *APOE* ε2/ε3 (*n* = 4), ε3/ε3 (*n* = 13), ε3/ε4 (*n* = 24) and ε4/ε4 (*n* = 12) genotype, we also observed differences in the levels of plasma apoE (ANOVA, *p* = 0.038), specifically between males with the *APOE* ε2/ε3 and ε3/ε4 genotype (Fig. 1c). Although levels of total plasma apoE varied between females with different *APOE* genotypes, and similarly between males with different *APOE* genotypes, only female *APOE* ε3/ε4 subjects exhibited significantly different plasma apoE levels compared to their male counterparts. Specifically, *APOE* ε3/ε4 females exhibited higher plasma total apoE levels (females (*n* = 23) 55.9 ± 25.5 μg/mL, males (*n* = 24) 39.2 ± 19.1 μg/mL, *p* = 0.014, Student’s *t*-test), attributed to an increase in the levels of the apoE4 isoform (females (*n* = 23) 20.7 ± 8.2 μg/mL versus males (*n* = 24) 14.0 ± 5.5 μg/mL, *p* = 0.002, Student’s *t*-test). Importantly, only in females, plasma apoE was significantly associated with age (Spearman’s (*ρ*) = 0.349, *p* = 0.003, *n* = 71).

### Plasma apoE isoform composition in *APOE* heterozygotes

**Fig. 2:**
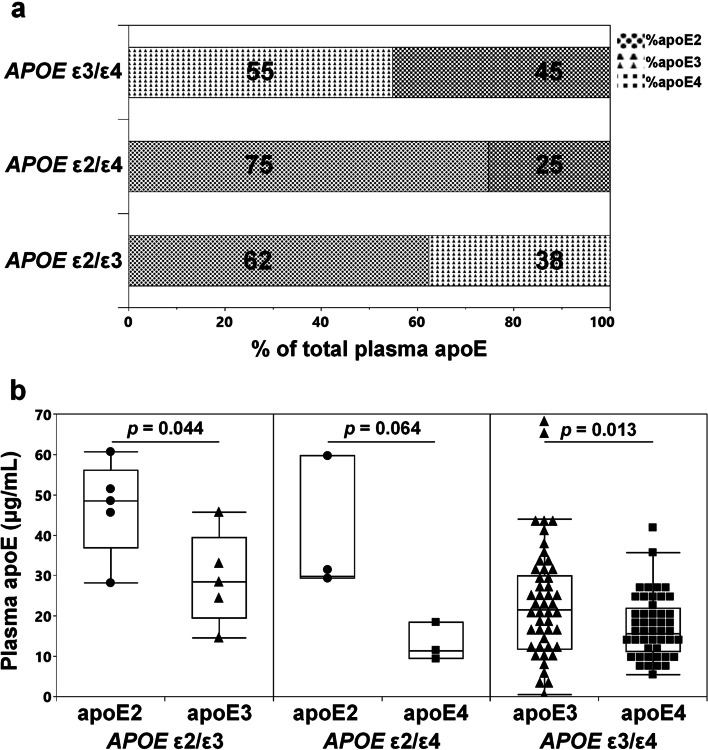
Total plasma apoE isoform distribution in *APOE* heterozygous individuals. Percentage (%) (**a**) and actual concentrations (**b**) of apoE2, apoE3, and apoE4 isoforms of total plasma apoE in *APOE* ε2/ε3, *APOE* ε2/ε4, and *APOE* ε3/ε4 subjects. Data are presented as average (**a**) or median (minimum–maximum) (**b**). *p*-values for *APOE* ε2/ε3 (black dots for apoE2, black triangles for apoE3), for *APOE* ε2/ε4 (black dots for apoE2, black squares for apoE4) and *APOE* ε3/ε4 (black triangles for apoE3, black squares for apoE4) were acquired using the Student’s *t*-test

With varying total apoE plasma levels between *APOE* genotypes we aimed to determine the contribution of the individual apoE isoforms to the total plasma apoE levels in *APOE* heterozygotes. In subjects with the *APOE* ε2/ε3 and *APOE* ε2/ε4 genotype, plasma total apoE consisted predominantly of the apoE2 isoform (62 ± 5% and 75 ± 2%, respectively) (Fig. 2a) with significantly different isoform levels in the *APOE* ε2/ε3 subjects (Fig. 2b). In subjects with the *APOE* ε3/ε4 genotype, the levels of apoE4 were nearly 30% lower than the apoE3 levels (Fig. 2a, b).

### Plasma apoE levels by diagnostic group and A/T/N classification

**Fig. 3:**
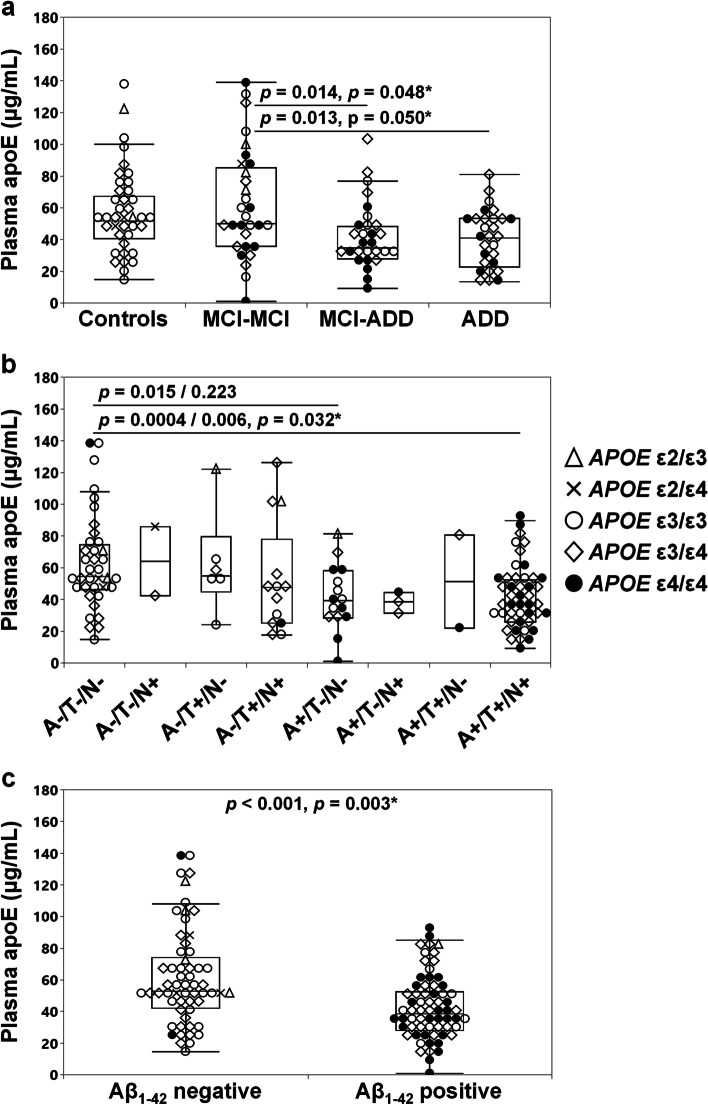
Plasma apoE levels per diagnostic group. Plasma apoE levels in controls, MCI-MCI, MCI-ADD and ADD patients (**a**) and in groups based on the A/T/N classification (**b**) and the Aβ_1-42_ status (**c**). Data is presented as median (minimum–maximum). Group differences were assessed using ANOVA (Tukey HSD post hoc) (**a**), the Kruskal–Wallis test followed by the Dunn’s test uncorrected/corrected for multiple comparisons using Bonferroni correction (**b**), or Mann–Whitney *U* test (**c**). Star marked *p*-values obtained after accounting for the *APOE* genotype of the studied subjects. The A-/T-/N + and A + /T + /N- groups were excluded from the statistical analysis due to low n-numbers (*n* = 2, in each group)

Plasma total apoE levels varied significantly between the diagnostic groups (Fig. 3a, ANOVA, *p* = 0.002). In detail, plasma total apoE levels were 1.5 times lower in MCI-ADD and ADD patients compared stable MCI patients respectively (Fig. 3a) with the difference remaining when accounting for *APOE* genotype (Wald Chi-Square *p* = 0.022). When combined, the prodromal and baseline ADD patients (*n* = 56) had 25% and 23% lower total plasma apoE levels compared to controls (ANOVA, *p* < 0.001, Tukey HSD post hoc, *p* = 0.012, *n* = 39) and stable MCI (Tukey HSD, *p* = 0.001, *n* = 30) respectively. When accounting for *APOE* genotype, a significant difference remained only between baseline ADD patients and stable MCI (Wald Chi-Square *p* = 0.008, Bonferroni post hoc, *p* = 0.007).

Accounting for AD brain pathology rather than clinical diagnosis, we compared plasma apoE levels among subjects classified according to the A/T/N classification system [50]. Among the resulting eight groups (A-/T-/N-, A-/T-/N + , A-/T + /N-, A-/T + /N +, A+/T-/N-, A + /T-/N + , A + /T + /N-, A + /T + /N +), we found a significant difference in the total plasma apoE levels (Kruskal Wallis, *p* = 0.007). The recorded difference between the “all pathology positive” (A + /T + /N +) and the “all pathology negative” (A-/T-/N-) groups remained significant when accounting for multiple comparisons (*n* = 15), and specifically, the Aβ_1-42_ positive subjects (with CSF Aβ_42_ levels lower than 630 pg/mL [47]) classified as A + /T-/N- and A + /T + /N +) exhibited lower plasma apoE levels compared to the “all pathology negative” subjects (A-/T-/N-) group (Fig. 3b). Irrespective of *APOE* genotype, Aβ_1-42_ positive subjects had 28% lower total plasma apoE levels compared to Aβ_1-42_ negative subjects (Fig. 3c). Accounting for the *APOE* genotype, plasma apoE levels remained different between A-/T-/N- and A + /T + /N + (Fig. 3b), as well as between Aβ_1-42_ positive and negative individuals (Fig. 3c). Subjects with A-/T-/N + and A + /T + /N- were excluded from the analysis due to the low sample numbers (*n* = 2 per group).

### Correlations between plasma apoE, apoE isoforms, cognition, and CSF markers

In the whole cohort, irrespective of diagnosis and *APOE* genotype, higher plasma apoE levels were significantly associated with higher MMSE scores levels of CSF Aβ_42_ and CSF Aβ_42_/Aβ_40_ and lower levels of CSF t-tau and p-tau, as well as CSF NfL levels (Table 5), whereas no associations were found between plasma apoE, plasma Nfl, plasma, and CSF YKL-40 nor CSF Aβ_40_, α-synuclein and KLK6 levels (data not shown). The association between Aβ_42_, Aβ_42_/Aβ_40_ and t-tau with plasma apoE levels remained when accounting for *APOE* genotype (Table 5).

**Table 5:** Correlations between plasma apoE levels, cognition, and markers in plasma and CSF

			*APOE* genotype unaccounted for		*APOE* genotype accounted for	
Group	**Markers**	**Samples (*n*)**	**Correlation**	***p*-value**	**Partial correlation**	***p*-value**
Whole cohort	MMSE scores	125	*ρ* = 0.263	0.003	*r*(122) = 0.168	0.062
	CSF Aβ_42_ (pg/mL)	119	*ρ* = 0.340	< 0.001	*r*(116) = 0.220	0.017
	CSF Aβ_42_/Aβ_40_	109	*ρ* = 0.349	< 0.001	*r*(106) = 0.242	0.012
	CSF t-tau (pg/mL)	120	*ρ* = − 0.322	< 0.001	*r*(117) = − 0.212	0.021
	CSF p-tau (pg/mL)	120	*ρ* = − 0.221	0.016	*r*(117) = − 0.121	0.190
	CSF NfL (pg/mL)	94	*ρ* = − 0.227	0.028	*r*(91) = − 0.203	0.051
Controls	Plasma NfL (pg/μL)	39	*ρ* = − 0.335	0.037	*r*(36) = − 0.328	0.044
MCI-MCI	CSF Aβ_42_ (pg/mL)	30	*r* = 0.467	0.009	*r*(27) = 0.398	0.033
MCI-ADD	CSF t-tau (pg/mL)	28	*r* = − 0.515	0.005	*r*(25) = − 0.403	0.037
	CSF p-tau (pg/mL)	28	*r* = − 0.436	0.020	*r*(25) = − 0.293	0.139
	CSF α-synuclein (pg/mL)	27	*r* = − 0.414	0.032	*r*(24) = − 0.333	0.096
	CSF KLK6 (ng/mL)	28	*r* = − 0.495	0.007	*r*(25) = − 0.382	0.049

*MCI-MCI* amnestic mild cognitive impairment patients that did not progress to an ADD diagnosis after 2 years, *MCI-ADD* patients with mild cognitive impairment that after two years fulfilled the criteria for an ADD diagnosis, *MMSE* mini mental state examination, *Aβ*_*42*_ Amyloid-β42 peptide, *t-tau* Total tau, p-tau: tau phosphorylated at Threonine 181 residue, *NfL* Neurofilament light chain, *KLK6* kallikrein 6. Correlation analysis was performed using Pearson’s (*r)*, or Spearman’s (*ρ*) tests. Partial correlations were shown as (*r*(degrees of freedom)) and obtained after controlling for *APOE* genotype

Stratifying our analysis based on diagnosis the association between plasma apoE and the Aβ_42_/Aβ_40_ ratio was eliminated; however, we observed that plasma apoE levels were negatively associated with plasma NfL only in controls (Table 5). Plasma apoE was furthermore positively associated with CSF Aβ_42_ only in MCI-MCI patients and negatively associated with CSF t-tau and p-tau only in the aMCI patients that over the 24 months study period received an ADD diagnosis (Table 5). Furthermore, accounting for diagnostic group we found significant negative correlations between plasma apoE and both CSF α-synuclein and KLK6 levels only in converting aMCI patients (Table 5), with the significant association with KLK6 levels remaining when taking *APOE* genotype into account. Grouping together the MCI-ADD and baseline ADD patients, the significant correlation between plasma apoE and CSF α-synuclein (Pearson’s (*r*) = -0.297, *p* = 0.029, *n* = 54) was eliminated when accounting for *APOE* genotype (Partial correlation *r*(51) = -0.257, *p* = 0.063). No statistically significant correlations between plasma levels of apoE and KLK6 or CSF and plasma YKL-40 levels were observed.

Lastly, we assessed whether specifically the apoE3 and apoE4 isoforms in *APOE* ε3/ε4 individuals were linked to MMSE scores, CSF AD biomarker levels, Aβ_40_, and the Aβ_42_/Aβ_40_ ratio_,_ as well as CSF α-synuclein and both CSF and plasma levels of KLK6, YKL-40, and NfL. Among these different markers, only CSF α-synuclein and NfL levels exhibited a negative association with the apoE4 (α -synuclein: Pearson’s (*r*) =  − 0.294, *p* = 0.045, *n* = 47, NfL: Pearson’s (*r*) =  − 0.333, *p* = 0.041, *n* = 47), but not apoE3 (α -synuclein: Pearson’s (*r*) =  − 0.269, *p* = 0.067, *n* = 47, NfL: Pearson’s (*r*) =  − 0.315, *p* = 0.054, *n* = 47) isoform levels. In addition, in the *APOE* ε3/ε4 subjects, we identified a positive association between total plasma apoE levels with both apoE3 (Pearson’s (*r*) = 0.961, *p* < 0.001, *n* = 47) and apoE4 (Pearson’s (*r*) = 0.878, *p* < 0.001, *n* = 47) isoforms, which were further shown to be positively linked to each other (Spearman’s (*ρ*) = 0.724, *p* < 0.001, *n* = 47).

## Discussion

Few studies have assessed a direct connection between apoE protein levels and AD status, although plasma apoE-related disease-specific phenotypic traits have been investigated in various diseases such as Down syndrome [51], lung [52] and liver diseases [53], and in relation to suicide [54]. Importantly, the *APOE ε4* genotype also increases the risk of dementia with Lewy bodies (DLB) [55] and promotes the incidence of dementia in pure synucleinopathies [56]. A recent study of a Stockholm-based cohort revealed elevated CSF, but not plasma, apoE levels in Parkinson’s disease (PD) patients compared to healthy individuals [57]. Furthermore, despite two large meta-analyses showing no connection between the *APOE* ε4 allele and the risk of multiple sclerosis [58] or amyotrophic lateral sclerosis (ALS) [59], studies by Gelman and colleagues as well as Lacomblez and co-authors on patients with multiple sclerosis and ALS, respectively, revealed lower levels of serum apoE in both patient categories compared to healthy individuals [60], as well as a negative correlation between plasma apoE levels and survival [61]. Hence, the peripheral levels of apoE may indeed have implications for pathological processes in the brain despite not crossing the BBB [5].

In the current study, we determined the levels of total apoE and the apoE isoform composition in baseline plasma samples from a longitudinally followed Norwegian cohort of sporadic ADD and aMCI patients, as well as healthy controls. We specifically aimed to assess whether baseline plasma apoE levels were associated with levels of CSF AD biomarkers and disease progression over a period of 24 months. In agreement with previous studies [20, 21, 62, 63], we found that levels of plasma apoE were the highest in subjects with the *APOE* ε2/ε3 genotype and the lowest in individuals carrying one, or two ε4 alleles. The functional relevance of *APOE* ε4-linked lower plasma apoE levels remains unclear, though we have previously found that higher total plasma apoE levels, quantified by ELISA in a subset of the herein included subjects, appeared to be beneficial in relation to cognition and CSF AD biomarker levels [39].

Previous studies have documented that levels of apoE isoforms in the plasma vary in heterozygous individuals [20, 21, 26, 37]. In agreement, we found that in heterozygous carriers of the ε2 allele the apoE2 isoform is the predominant isoform in the plasma. Previous studies, including our own, found that the apoE3 isoform accounted for approximately 70% of the total apoE in plasma from *APOE* ε3/ε4 subjects [21, 37]. In our current cohort, we instead found an almost equal distribution between the apoE3 and apoE4 isoforms (55%/45%) in plasma from subjects with the *APOE* ε3/ε4 genotype. A similar ratio of apoE3 and apoE4 isoforms in plasma of *APOE* ε3/ε4 individuals was reported by Fukumoto and colleagues in 2003 [64]. The combined outcome of previous studies, however, is that in plasma from *APOE* ε3/ε4 subjects the apoE4 isoform exists in lower concentrations than its apoE3 counterpart.

Whether the difference between apoE2, apoE3, and apoE4 isoforms concentrations in *APOE* heterozygous individuals can be explained by differential expression of the different alleles still needs to be investigated. Previous studies have shown differences in total apoE mRNA expression in the brains of healthy and AD subjects with *APOE* ε2/ε4, *APOE* ε2/ε3, and *APOE* ε3/ε4 genotypes [65, 66]; it is however not clear whether similar differences exist in the liver. As suggested by previous studies, the apoE4 isoform may be prone to a higher degradation rate in plasma [67–70]; however, whether the degradation rate is affected by structural differences (monomeric versus dimeric apoE), post-translational modifications, or lipidation in combination with differences in LDLR affinities of the different apoE isoforms remains to be determined. Since the non-apoE4 isoforms are the dominant isoforms in plasma of *APOE* ε4 heterozygous individuals [20, 21, 26, 37], we speculate it may be beneficial as higher plasma apoE4 levels in our recent study of mice with humanized *APOE* ε4/ε4 livers was negatively associated with markers of synaptic integrity, neuroinflammation, and insulin signaling [71]. In addition, we previously showed that a higher plasma apoE4 to apoE3 ratio was linked to reduced grey matter volume and higher glucose hypometabolism in brain areas normally affected by AD in a cohort of cognitively healthy *APOE* ε3/ε4 individuals [37].

Results have been contradictory as to whether plasma apoE levels vary between healthy control subjects and AD patients [25, 62, 63]. Our own studies of AD patients and non-AD controls showed no difference in the levels of plasma apoE [21]. In contrast, in the present study, we documented lower levels of plasma apoE in AD patients that either entered the study with an ADD diagnosis at baseline, or who converted to ADD from aMCI over 24 months, compared to controls. However, this finding was mainly driven by the *APOE* ε4-carriers in these groups. On the contrary, the recorded difference in plasma apoE levels between stable MCI patients and those that converted to an ADD diagnosis appeared not to be due to differences in *APOE* ε4 frequency (chi-square, *p* = 0.33). Hence, our results are in agreement with a recent study demonstrating a link between lower plasma apoE levels and AD disease pathogenesis [63]. Of note, potential discrepancies in the outcome of different case–control studies may be due to technical reasons however we also speculate that plasma apoE levels may vary between populations. With the current study accounted for, our team has assessed plasma apoE levels in three different cohorts including non-demented *APOE*ε3/ε4 individuals from the Arizona *APOE* Study (USA) [37], non-AD subjects and AD patients from Sweden [21], and the current cohort of patients and controls from Norway. The average total plasma apoE levels in non-demented subjects of those studies ranged from approximately 35 μg/mL [37] to 25 μg/mL [21] and the current study of approximately 57 μg/mL. Other studies have with similar methodology assessed plasma apoE levels in subjects from Germany (LIFE-Adult Study, MCI cohort) [72] and USA (Familial AD subjects) [26]. Plasma apoE levels in the German cohort ranged between 0.82 and 1.31 μmol/L corresponding to approximately 28–45 μg/mL (using 34 kDa as molecular weight) [72]. In the study by Baker-Nigh et al., the authors calculated the apoE levels as a ratio of endogenous apoE to apoE internal standard rather than apoE concentrations which makes extrapolating the results to actual concentrations difficult [26]. Frequencies of the *APOE* ε4 allele vary across geographical locations with uncertain effect on apoE levels and AD prevalence. For example, an *APOE* ε4 “gradient” with higher frequency in the Northern European countries versus lower occurrence in the Southern European counties has been described however without a corresponding increase in the prevalence of cognitive dysfunction [73]. We speculate that lifestyle-associated factors including diet might modify the *APOE* ε4-driven risk of neurodegeneration, especially in the light of reported beneficial effects of fish and polyunsaturated fatty acid consumption on the risk of cognitive impairment [74] and AD [75]. Other low fat diets such as, the Mediterranean, Japanese, and plant-based diets have been shown to reduce cognitive decline, whereas a Western diet, characterized by elevated concentrations of sugars and fats (saturated and trans-fatty acids), as well as an overall higher glycemic index, was shown to have adverse effects on cognition and promoting a higher risk for dementia (reviewed in [76]). With our current cohort consisting of ethnic Norwegian, it is especially interesting to note that a beneficial connection between a Nordic diet and cognition [77, 78], especially in *APOE* ε4-carriers [79], was previously documented.

The *APOE* ε4 allele is generally considered to promote Aβ brain pathology at an earlier age even in individuals without cognitive symptoms [80]. Employing the A/T/N classification in the current cohort, we found that plasma apoE levels were lower in subjects with Aβ_1-42_ pathology but yet in the absence of neurodegeneration and tau-pathology. The lowest plasma apoE levels were found in subjects that were A + /T + /N + , and we acknowledge that our findings may be skewed by the size of the investigated groups. Future and larger cohort studies are needed to confirm our findings.

In addition to the conventional CSF AD biomarkers, CSF levels of α-synuclein appear altered in AD [81]. We specifically found that higher CSF α-synuclein levels were linked to Aβ pathology in *APOE* ε4-carrying subjects with asymptomatic familial AD [45], and to disease progression in sporadic AD patients examined in the current study. Moreover, the levels of the α-synuclein cleaving enzyme KLK6 were also altered in AD patients from the current cohort [44]. In addition, we here analyzed the fluid levels (plasma and CSF) of YKL-40, an astrocytic inflammatory marker previously shown to be upregulated in AD patients [82, 83]. Using multivariate correlation analyses, we assessed whether plasma apoE levels were related to α-synuclein, KLK6, and YKL-40 and found that in addition to significant associations with cognition (MMSE) and the CSF AD biomarkers (except for Aβ_40_), low levels of plasma apoE were related to higher CSF KLK6 and α-synuclein levels specifically in aMCI patients converting to ADD. We find the described negative association between the plasma apoE and CSF α-synuclein levels (absent when accounting for *APOE* genotype) very intriguing especially when considering the frequently reported co-occurrence of AD and Lewy body pathology (reviewed in [84, 85]) and the recently reported association between the *APOE* ε4 allele and α-synuclein pathology [86, 87]. Elaborating on the potential implication of an association between plasma apoE and specifically the apoE4 isoform levels and CSF α-synuclein levels is difficult especially since it is not clear what altered CSF α-synuclein de facto reflect on. Earlier studies have documented higher CSF α-synuclein levels in AD patients whereas patients with synucleinopathies were reported to have lower CSF α-synuclein levels [88–92]. In addition, we found a positive association between higher CSF α-synuclein levels and PET-verified Aβ-pathology in asymptomatic familial AD patients who carried the *APOE* ε4 allele [45], after symptom onset the direction of this association was inversed. If lower levels of α-synuclein pathology mirror Lewy pathology in the brain (as seen in synucleinopathy patients), then higher plasma apoE4 levels would indeed be associated with more α-synuclein pathology in the MCI-ADD subjects in the current cohort. Further studies are needed to test this assumption.

As shown, low plasma apoE levels may be related to various aspects of AD pathology which is further illustrated by our finding that low plasma apoE levels were linked to higher plasma and CSF NfL levels, previously shown to increase in AD [93, 94] and other neurodegenerative disorders [95]. Intriguingly, the observed associations between plasma apoE with CSF NfL (whole cohort) or plasma NfL (control subjects only) were not consistent and not present in the separate diagnostic groups, potentially due to lack of statistical power. Overall, an inverse connection between plasma apoE specifically with CSF NfL levels, even if only in controls, points to a beneficial effect of higher plasma apoE levels in terms of neurodegeneration. Extrapolating those results, they may be in agreement with the previously proposed beneficial effect of higher plasma apoE on AD risk [24]. Although it may seem more logical for CSF apoE rather than plasma apoE to be correlated with brain pathology and CSF markers thereof, *APOE* ε4 status and cognitive decline, previous studies have indeed shown that CSF apoE levels are not affected by these parameters [20, 21, 27]. We speculate that rather than the CSF apoE protein levels per se, other features like structure and post-translational modifications (not assessed in the current study) as well as potential differences in the regulation of the two separate pools of apoE (plasma versus CSF) may attribute disease-relevant associations to plasma rather than CSF apoE levels despite the notion that plasma apoE does not cross the BBB [5].

Lastly, we addressed a potential interaction between sex and plasma apoE levels as previous results have been inconsistent [21, 37, 96–98]. We found no difference in the total plasma apoE levels between males and females, but, *APOE* genotype stratification revealed higher plasma apoE levels in female *APOE* ε3/ε4-carriers versus their male counterparts, similar to our previous findings [37]. Females are at higher risk of AD compared to males [99] and interactions between sex and *APOE* have also been described (reviewed in [100]). Hence, we speculate that the risk of AD in females may be modulated by plasma apoE levels driven by *APOE* genotype.

## Limitations

The main limitation of this study is the limited number of study subjects which reduces the overall statistical power. As a consequence, the number of individuals in each group categorized according to the ATN classification was small, further limiting our statistical power and complicating our analysis of potential changes in plasma apoE levels based on Aβ (A) and/or tau (T) pathology and neurodegeneration (N). Similarly, the lack of significant differences in plasma apoE3 versus apoE4 isoform levels in *APOE* ε3/ε4 subjects (second most common *APOE* genotype) with different disease status we speculate could be due to the limited sample size. Our cohort further included only eight *APOE* ε2 positive individuals whereof none were homozygous, mainly due to the in general low frequency of this genotype. Last, we were unable to test a potential influence of the body mass index (BMI), or cardiovascular risk factors, such as hypercholesterolemia, on plasma apoE levels and cannot rule out an effect of these although our previous findings in healthy *APOE* ε3/ε4 subjects suggested that there are no differences in plasma apoE levels between individuals with a pathological (> 25) versus normal BMI [101].

## Conclusion

In summary, we here reported the total plasma apoE and apoE isoform levels in patients in the AD continuum versus control subjects using an MS-based assay. We confirmed that the *APOE* ε4 genotype was associated with lower plasma apoE levels and further that patients with prodromal and manifest ADD exhibited the lowest levels. Moreover, we corroborated that low plasma apoE levels were unfavorably linked to cognition and CSF AD biomarkers and that Aβ_1-42_ pathology positive subjects, as assessed using the A/T/N classification system, exhibited lower plasma apoE. In addition, we found no link between plasma apoE and levels of the astrocytic inflammatory marker YKL-40 in the CSF; however, we demonstrated novel associations between low apoE levels and higher levels of both KLK6 and α-synuclein, specifically in aMCI patients converting to ADD. Lastly, when accounting for the *APOE* genotype the associations between plasma apoE, CSF AD biomarkers, cognition, and disease status remained significant. Together, these findings suggest that the recorded associations between plasma apoE levels and markers of brain pathological processes could not be explained by *APOE* genotype. The biological and functional relevance of altered levels of apoE in plasma to processes in the brain needs to be addressed in future studies.

## Supplementary Information

**Additional file 1:** **Supplementary Table 1.** Spiked amount of heavy labelled peptides in each sample. **Supplementary Table 2.** Endogenous peptides identified in the different *APOE* genotypes. **Supplementary Fig. 1.** Quantification of apoE isoforms. Formula used for the quantification of endogenous apoE peptide levels (LGPLVEQGR, LAVYQAGAR, LGADMEDVCR, LGADEMDVR and CLAVYQAGAR) (a). Linear regression of peptide LAVYQAGAR, with the peptide LGPLVEQGR in subjects with *APOE* ε3/ε3 (open dots), *APOE* ε3/ε4 (open rhombus) and *APOE* ε4/ε4 (black dots) genotype (b). Linear regression between apoE3 levels determined by the common apoE2/3 peptide LGADMEDVCGR and calculated by subtracting the apoE4 peptide LGADMEDVR from the common apoE3/4 peptide LAVYQAGAR (c). Plasma apoE4 levels as determined by the apoE4 specific peptide LGADMEDVR and calculated by subtracting the concentration of the apoE2/3 peptide LGADMEDVCGR from the apoE3/4 peptide LAVYQAGAR (d). Linear regression between plasma apoE levels calculated by use of the different isoform-specific peptides and the apoE isoform common peptide LGPLVEQGR (e) in individuals with *APOE* ε2/ε3 (open triangles), *APOE* ε2/ε4 (x-shape), *APOE* ε3/ε3 (open dots), *APOE* ε3/ε4 (open rhombus) and *APOE* ε4/ε4 (black dots). **Supplementary Fig. 2.** Calibration curves generated for the apoE digested peptides LGPLVEQGR (a), LGADMEDVCGR (b), LAVYQAGAR (c) and CLAVYQAGAR (d). Graph illustrates the area response ratio of the heavy labelled peptide to the endogenous variant plotted against the increasing amount of the heavy labelled peptide. Axes are illustrated in logarithmic scale for better separation of the data points that did not undergo log transformation. Calibration curves were generated using the weighted sum of squares (1/X^2^).

## Data Availability

Not applicable.

## Abbreviations

A/T/N: Amyloid, Tau and Neurodegeneration
AD: Alzheimer’s disease
ADD: Alzheimer’s disease dementia
AGC: Automatic gain control
ALS: Amyotrophic lateral sclerosis
aMCI: Amnestic mild cognitive impairment
ANCOVA: Analysis of covariance
apoE: Apolipoprotein E
Arg: Arginine
Aβ_40_: Amyloid-β40
Aβ_42_: Amyloid-β42
BBB: Blood–brain-barrier
BMI: Body mass index
CNS: Central nervous system
CSF: Cerebrospinal fluid
Cys: Cysteine
DLB: Dementia with Lewy bodies
DTT: Dithiothreitol
EDTA: Ethylenediaminetetraacetic acid
ELISA: Enzyme-linked immunosorbent assay
IAA: Iodoacetamide
KLK6: Kallikrein 6
LC: Liquid chromatography
LDLR: Low-density lipoprotein receptor
MCI: Mild cognitive impairment
MMSE: Mini-mental-state-examination
MS: Mass spectrometry
NfL: Neurofilament light chain
PD: Parkinson’s disease
p-tau: Phosphorylated tau at Threonine (Thr) 181
TFA: Trifluoroacetic acid
t-tau: Total tau
