# Supplementary material for: Plasma apolipoprotein E levels in longitudinally followed patients with mild cognitive impairment and Alzheimer’s disease
Source: Alzheimers Res Ther. 2022 Aug 24;14:115. doi: 10.1186/s13195-022-01058-9 (PMC9400269; doi:10.1186/s13195-022-01058-9)
Supplement: Supplementary file 1 — Additional file 1: Supplementary Table 1. Spiked amount of heavy labelled peptides in each sample. Supplementary Table 2. Endogenous peptides identified in the different APOE genotypes. Supplementary Fig. 1. Quantification of apoE isoforms. Formula used for the quantification of endogenous apoE peptide levels (LGPLVEQGR, LAVYQAGAR, LGADMEDVCR, LGADEMDVR and CLAVYQAGAR) (a). Linear regression of peptide LAVYQAGAR, with the peptide LGPLVEQGR in subjects with APOE ε3/ε3 (open dots), APOE ε3/ε4 (open rhombus) and APOE ε4/ε4 (black dots) genotype (b). Linear regression between apoE3 levels determined by the common apoE2/3 peptide LGADMEDVCGR and calculated by subtracting the apoE4 peptide LGADMEDVR from the common apoE3/4 peptide LAVYQAGAR (c). Plasma apoE4 levels as determined by the apoE4 specific peptide LGADMEDVR and calculated by subtracting the concentration of the apoE2/3 peptide LGADMEDVCGR from the apoE3/4 peptide LAVYQAGAR (d). Linear regression between plasma apoE levels calculated by use of the different isoform-specific peptides and the apoE isoform common peptide LGPLVEQGR (e) in individuals with APOE ε2/ε3 (open triangles), APOE ε2/ε4 (x-shape), APOE ε3/ε3 (open dots), APOE ε3/ε4 (open rhombus) and APOE ε4/ε4 (black dots). Supplementary Fig. 2. Calibration curves generated for the apoE digested peptides LGPLVEQGR (a), LGADMEDVCGR (b), LAVYQAGAR (c) and CLAVYQAGAR (d). Graph illustrates the area response ratio of the heavy labelled peptide to the endogenous variant plotted against the increasing amount of the heavy labelled peptide. Axes are illustrated in logarithmic scale for better separation of the data points that did not undergo log transformation. Calibration curves were generated using the weighted sum of squares (1/X2). [file 13195_2022_1058_MOESM1_ESM.docx]

**SUPPLEMENTARY INFORMATION**

**Plasma apolipoprotein E levels in longitudinally followed patients with mild cognitive impairment and Alzheimer’s disease**

Andreas Giannisis^a^, Asma Al-Grety^b^, Henrik Carlsson^b^, Kalicharan Patra^a^, Daniel Twohig^a^, Sigrid Botne Sando^c,d^, Camilla Lauridsen^d^, Guro Berge^d^, Gøril Rolfseng Grøntvedt^c^, Geir Bråthen^c,d^, Linda R. White^c,d^, Kim Kultima^b^ , Henrietta M. Nielsen^a,^ *

^a^*Department of Biochemistry and Biophysics, Stockholm University*

^b^*Department of Medical Sciences, Clinical Chemistry, Uppsala University, Uppsala, Sweden.*

^c^*Department of Neurology, University Hospital of Trondheim, Trondheim, Norway*

^d^*Department of Neuromedicine and Movement Science (INB), Norwegian University of Science and Technology (NTNU), Faculty of Medicine and Health Sciences, Trondheim, Norway*

*Correspondence: [henrietta.nielsen@dbb.su.se](mailto:henrietta.nielsen@dbb.su.se)

The supplementary information include two tables (Supplementary Table 1-2), and two figures (Supplementary Fig. 1-2).

**Supplementary Table 1:** Spiked amount of heavy labelled peptides in each sample.

| Internal standards | ApoE isoform | Spiked amount (fmoles) |
| --- | --- | --- |
| LGPLVEQGR | apoE2, apoE3 and apoE4 | 175 |
| LAVYQAGAR | apoE3 and apoE4 | 175 |
| CLAVYQAGAR | apoE2 | 175 |
| LGADMEDVCGR | apoE2 and apoE3 | 175 |
| LGADMEDVR | apoE4 | 105 |

apoE: apolipoprotein E.

**Supplementary Table 2:** Endogenous peptides identified in the different *APOE* genotypes.

| *APOE* genotype | Endogenous peptide |
| --- | --- |
| *APOE* ε2/ε3 | LAVYQAGAR, CLAVYQAGAR & LGADMEDVCGR |
| *APOE* ε2/ε4 | LAVYQAGAR, CLAVYQAGAR, LGADMEDVCGR & LGADMEDVR |
| *APOE* ε3/ε4 | LAVYQAGAR, LGADMEDVCGR & LGADMEDVR |
| *APOE* ε3/ε3 | LAVYQAGAR & LGADMEDVCGR |
| *APOE* ε4/ε4 | LAVYQAGAR & LGADMEDVR |

*APOE*: Apolipoprotein E gene.

**
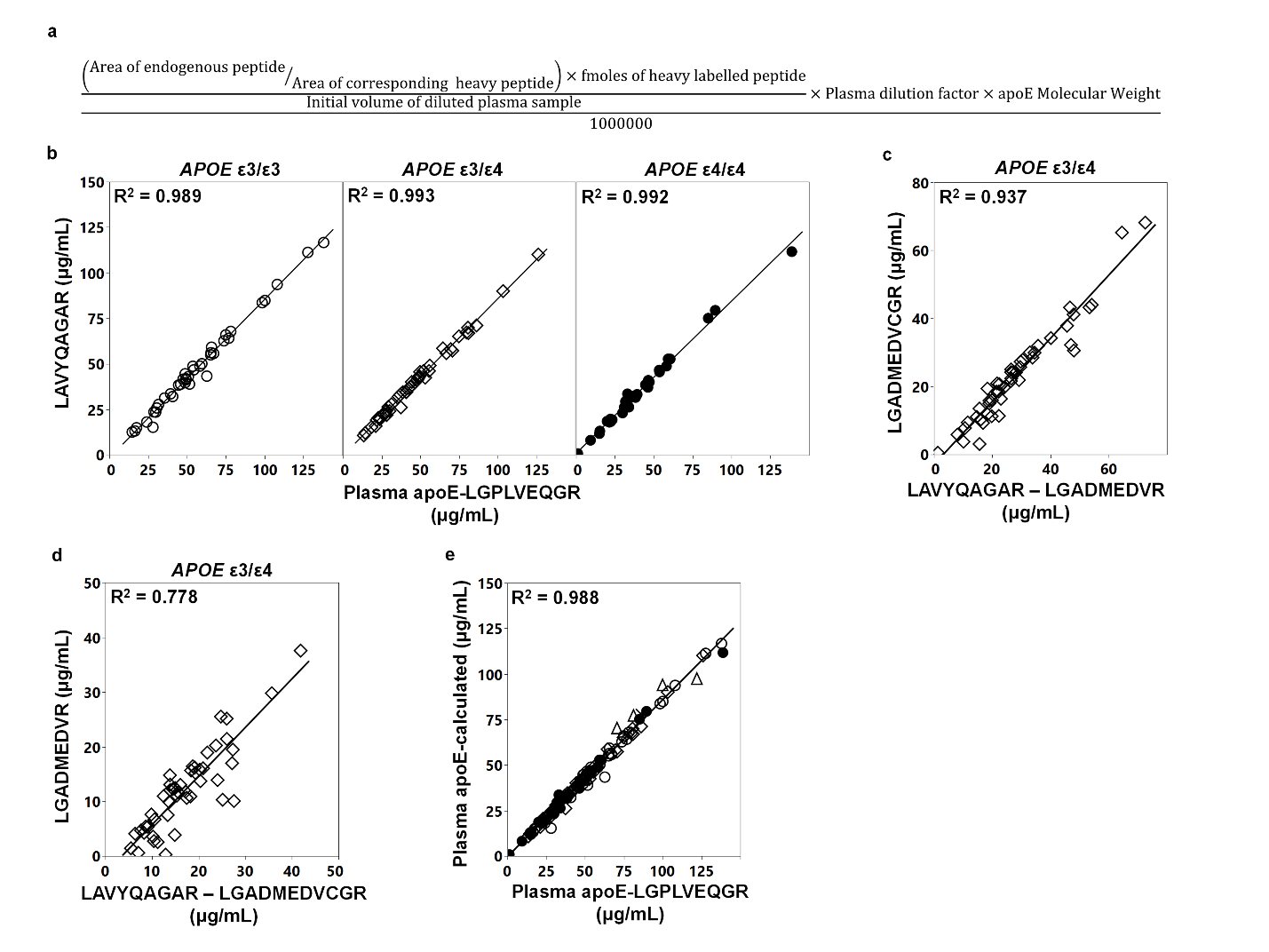
**

**Supplementary Fig. 1. Quantification of apoE isoforms.** Formula used for the quantification of endogenous apoE peptide levels (LGPLVEQGR, LAVYQAGAR, LGADMEDVCR, LGADEMDVR and CLAVYQAGAR) (**a**). Linear regression of peptide LAVYQAGAR, with the peptide LGPLVEQGR in subjects with *APOE* ε3/ε3 (open dots), *APOE* ε3/ε4 (open rhombus) and *APOE* ε4/ε4 (black dots) genotype (**b**). Linear regression between apoE3 levels determined by the common apoE2/3 peptide LGADMEDVCGR and calculated by subtracting the apoE4 peptide LGADMEDVR from the common apoE3/4 peptide LAVYQAGAR (**c**). Plasma apoE4 levels as determined by the apoE4 specific peptide LGADMEDVR and calculated by subtracting the concentration of the apoE2/3 peptide LGADMEDVCGR from the apoE3/4 peptide LAVYQAGAR (**d**). Linear regression between plasma apoE levels calculated by use of the different isoform-specific peptides and the apoE isoform common peptide LGPLVEQGR (**e**) in individuals with *APOE* ε2/ε3 (open triangles), *APOE* ε2/ε4 (x-shape), *APOE* ε3/ε3 (open dots), *APOE* ε3ε4 (open rhombus) and *APOE* ε4/ε4 (black dots).

**
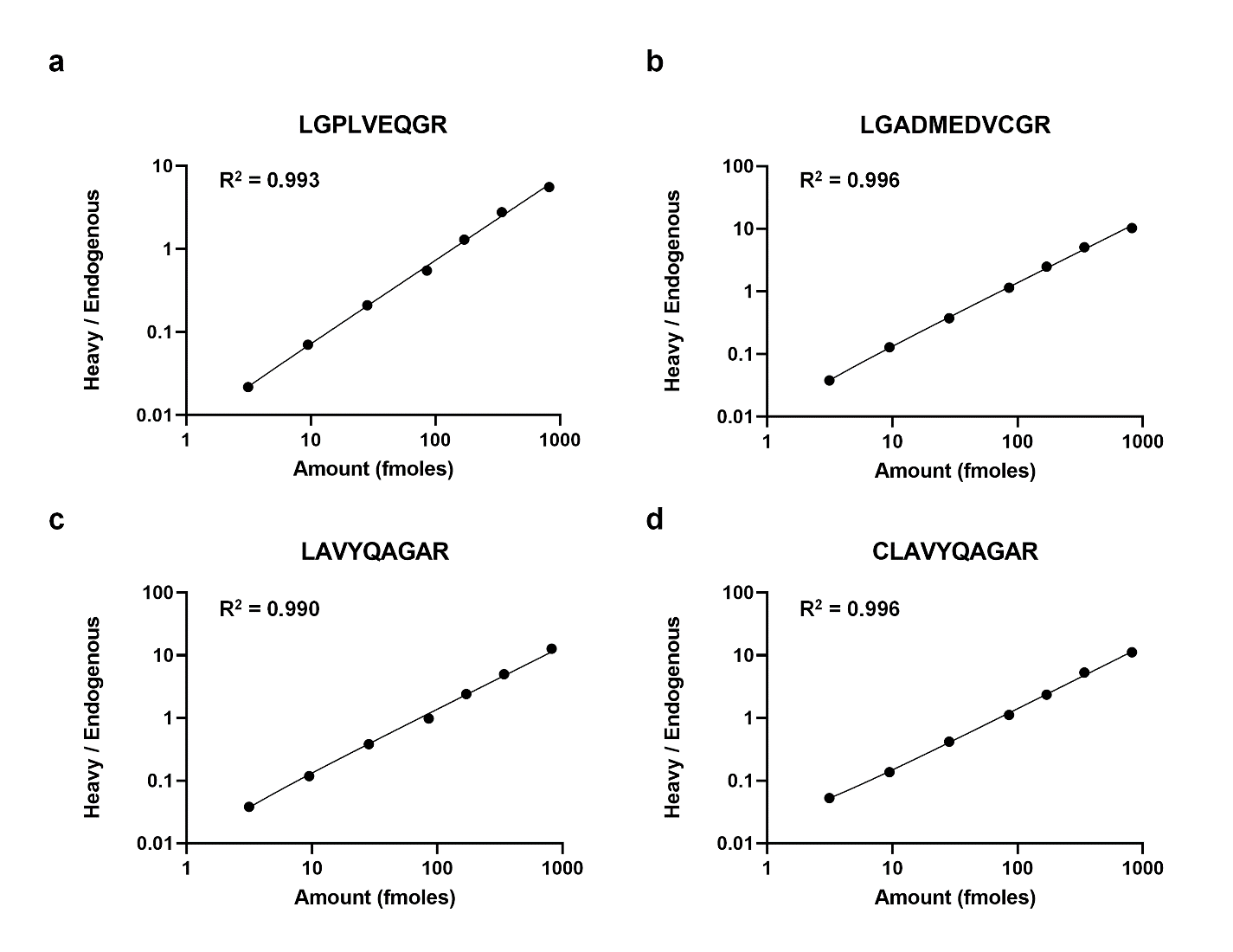
**

**Supplementary Fig. 2. Calibration curves generated for the apoE digested peptides LGPLVEQGR (a), LGADMEDVCGR (b), LAVYQAGAR (c) and CLAVYQAGAR (d).** Graph illustrates the area response ratio of the heavy labelled peptide to the endogenous variant plotted against the increasing amount of the heavy labelled peptide. Axes are illustrated in logarithmic scale for better separation of the data points that did not undergo log transformation. Calibration curves were generated using the weighted sum of squares (1/X^2^).
